# Interrelationships between mentalizing capacity, symptoms of depression and anxiety, and coparenting in parents of school aged children

**DOI:** 10.3389/fpsyg.2026.1481550

**Published:** 2026-05-20

**Authors:** Mia De Palma, Elizabeth Izett, Rosanna Rooney, Maryanne McDevitt, Vincent Mancini

**Affiliations:** 1 School of Population Health, Faculty of Health Sciences, Curtin University, Perth, WA, Australia; 2 Telethon Kids Institute, University of Western Australia, Nedlands, WA, Australia

**Keywords:** anxiety, coparenting, depression, parent mental health, reflective functioning

## Abstract

**Introduction:**

Mentalization (the ability to hold in mind and interpret one’s own and others’ mental states in order to make sense of behaviour) has been consistently linked with the development of secure parent-infant attachment as well as with adult mental health. A smaller body of research has begun to examine how mentalizing capacity may be linked with coparenting (parents’ ability to work together for the wellbeing of their child), which is another factor consistently linked with better child outcomes, as well as lower levels of anxiety and depression and greater overall wellbeing in parents. Reflective functioning is an aspect of mentalization that involves the outward reflection of the mental states of the self and others.

**Methods:**

The present study aimed to cross-sectionally investigate interrelationships between mentalizing capacity measured by general reflective functioning (RF) scores, parental mental health and coparenting quality among couples. Participants were 82 coupled parents of a child aged 3 years to 17 currently involved in schooling who completed an online Qualtrics questionnaire.

**Results:**

Results indicate that poorer RF was related to reduced coparenting quality in mothers, while symptoms of depression and anxiety were related to worse coparenting quality in fathers. Parental depression and anxiety was also associated with reduced RF. Among fathers, we also found an indirect effect of RF on coparenting relationships via symptoms of depression and anxiety.

**Discussion:**

These findings add to a new field of inquiry demonstrating the important role mentalization plays in both parental anxiety and depression and the coparenting relationship, factors all shown to be crucial for ongoing child development and wellbeing.

## Introduction

Reflective functioning in parents is an ability that has the potential to influence a number of parenting and relationship aspects. The capacity to make sense of mental states (e.g., emotions, thoughts, motivation) and how they impact behaviour in oneself and others is a skill that underpins our ability as human beings to function well in social and emotional contexts (Borelli et al., 2020). Research concerning mental states initially grew within the field of philosophy, garnering wider interest as developmental psychologists began to examine how theory of mind first develops in young children (Ensink and Mayes, 2010). This topic has been more recently adopted by psychodynamic theorists under the label of ‘*mentalizing*’, or in its operationalized form, ‘*reflective functioning*’ (RF) (Fonagy, 1991; Fonagy et al., 1991). Reflective functioning refers to an individual’s ability to hold another person’s mental state in mind and reflect that mental state back to the other person (Fonagy and Target, 1997).

The capacity to mentalize is thought to develop within the context of a secure attachment relationship in which a parent (a term inclusive of any primary caregiver) is able to make sense of, and mirror back, the mental states of their child, without themselves becoming overwhelmed (Fonagy and Target, 1997). A child who experiences this emotional availability from their parents is more able to securely explore the world around them and make sense of their experiences and emotions, giving them the resources to develop the capacity to regulate their emotions independently (Fonagy and Target, 1997). These children are more likely to develop a coherent sense of self and may go on to experience better social emotional wellbeing later in life (Benbassat and Priel, 2012; Camoirano, 2017; Gordo et al., 2020; Salo et al., 2021). However, the capacity for children to engage in this process is contingent on their parent’s own mentalizing capacity.

Mentalizing capacity can be impaired by many factors including a parents’ own history of relational trauma and poor mental health (van Rensburg et al., 2023; Georg et al., 2023). When a parent or their child is in a state of heightened emotional arousal, parents may be particularly vulnerable to losing a sense of their own and their child’s internal world (Borelli et al., 2020). When this happens, parents may enter a state in which their capacity to mentalize is temporarily impaired. Parents with strong RF are more able to accurately make sense of their child’s behaviour, and regulate their own responses in stressful times in order to remain emotionally present and provide emotionally attuned responses to their children. In the parenting relationship, mentalizing can also refer to each person’s ability to hold their partner’s mental state in mind.

Given the role that mentalizing capacity plays in both emotional regulation and interpersonal relationships, it is not surprising that underdeveloped or absent mentalizing has been found to play an important role in an individual’ s own mental health (Luyten et al., 2013; Luyten and Fonagy, 2018). For example, poor mentalizing has been linked with psychopathology including depression (Belvederi Murri et al., 2017; Bigelow et al., 2018; Fischer-Kern et al., 2013; Wendelboe et al., 2021), eating disorders (Jewell et al., 2016), conduct disorder (Taubner et al., 2019), substance use disorders, psychosis (Bateman and Fonagy, 2019), as well as personality disorders including borderline personality disorder (Badoud et al., 2018; Fonagy and Bateman, 2007), antisocial personality disorder (Bateman et al., 2019), avoidant personality disorder and narcissistic personality disorders (Simonsen and Euler, 2019). Studies have found that interventions targeting RF can reduce levels of distress across a range of psychological disorders (Hayden et al., 2018). This research highlights the significant role mentalizing capacity plays in maintaining mental health, with research suggesting that this remains true within community samples of parents (Ierardi et al., 2022).

Given the large body of research demonstrating links between RF and mental health as well as parent-infant attachment relationships and later child wellbeing, a newer field of research has begun to examine how RF plays out within other relationships (De Palma et al., 2023). In particular, researchers are beginning to examine the role that RF may play in couple relationships, including couples who are also parents (Borelli et al., 2020). The dynamic between parents is important to consider given the impact the parental couple relationship plays on ongoing child development and social emotional wellbeing (Cowan and Cowan, 2002; Feinberg, 2003; Harold and Leve, 2012; Salo et al., 2022; Yap et al., 2014). A complementary body of evidence has also started to explore interactions between RF and *coparenting*, a similar but distinct concept that has its roots within family systems theory (De Palma et al., 2023). Coparenting can be defined as adults sharing the responsibility for taking care of children (Cabrera et al., 2012).

We will now consider the influence of parents’ RF on coparenting. As couples become parents, an added dimension enters their relationship as they navigate their new shared role (Salo et al., 2021). Each parent enters a coparenting relationship with their own history of being parented, and this may come with different opinions about how best to raise their child (Borelli et al., 2020). A successful coparenting partnership forms when parents are able to navigate these separate histories and opinions alongside their romantic relationship, and come together in harmony for the benefit of their child (Salo et al., 2021). A large body of research has demonstrated links between coparenting and child outcomes such as emotional wellbeing (Teubert and Pinquart, 2010; Umemura et al., 2015), social and cognitive development and academic achievement (Cabrera et al., 2012; Cheng et al., 2009; Nandy et al., 2021; Shai, 2019). Some researchers have argued that family level processes, and coparenting in particular, have a greater impact on child development than both individual parenting abilities and the couple relationship (Boričević Maršanić and Kušmić, 2013; Feinberg and Kan, 2008). This is because each relationship within a family system is interdependent, and the coparenting relationship in particular takes into account triadic family level interactions, which offers more insight into family level processes than the dyadic interactions seen within the parent–child relationship or the couple relationship (Boričević Maršanić and Kušmić, 2013). A poor coparenting alliance has also been found to negatively impact parental mental health (Cabrera et al., 2012), though this relationship may be bidirectional, with other research suggesting that poor mental health may lead to a decline in the coparenting relationship (Turney and Hardie, 2021; Williams, 2018). Given these relationships between RF, mental health and child wellbeing, new research has begun to explore how the couple relationship and coparenting in particular may fit within this picture (De Palma et al., 2023).

Recent research has found links between mentalizing capacity and both relationship satisfaction (Borelli et al., 2021; Salo et al., 2022) and improved coparenting relationships (De Palma et al., 2023; Holtzinger, 2021; Jessee, 2012; Marcu et al., 2016; Shai et al., 2017). This makes sense from a theoretical perspective, given greater mentalizing capacity should allow an individual to better consider the thoughts and feelings that underlie their partner’s behaviour. In turn, this may reduce conflict, and better enable each partner to meet the other’s needs and communicate more effectively (Borelli et al., 2020).

Despite links between mentalizing capacity, parental mental health and coparenting, very few studies have considered these variables together. Mazzeschi et al. (2019) found that parents of children with attention deficit/hyperactivity disorder (ADHD) displayed poorer RF, increased symptoms of depression, and worse coparenting quality (in mothers only) than parents whose children did not have ADHD (Mazzeschi et al., 2019).

Dollberg et al. (2021) also examined these variables together, finding an indirect effect of parental anxiety on child outcomes via parental coparenting quality, however RF was not found to moderate this relationship (Dollberg et al., 2021). Only when coparenting was not included in the model was the relationship between parental anxiety and child outcomes moderated by RF (Dollberg et al., 2021). De Palma et al. (2023) also examined these variables together, finding that parental depression and anxiety predicted the coparenting relationship. This study further found an indirect effect of negative coparenting on child development via parental reflective functioning (De Palma et al., 2023). However this research did not recruit couples, which may have impacted findings, given different views each parent may have regarding the coparenting relationship and child development (De Palma et al., 2023). Overall, prior research has consistently found worse RF among those experiencing poor mental health (Luyten and Fonagy, 2018), while higher RF has been linked with more effective coparenting (Holtzinger, 2021). Parental depression and anxiety have also been linked with poorer coparenting (De Palma et al., 2023). However few studies have investigated the interrelationship between these variables among couples. Based on the past research, we will test the mediating effect of parent anxiety and depression on the link between reflective functioning and the coparenting relationship. It appears that reflective functioning impacts mental health, and mental health affects coparenting; however, the nature of this relationship has not yet been tested.

## The current study: aims and hypotheses

The present study aims to fill this gap, by cross-sectionally investigating how reflective functioning, parental mental health and coparenting are linked among couples. This is important given the scarcity of research examining these variables together, with few models available that consider potential interrelationships between one parent’s functioning on the outcomes of their partner. In this study, we measure general reflective functioning in parents as opposed to the more specific concept of parental reflective functioning, which relates to the use of reflective functioning in relation to parenting their children only.

We hypothesised that:

1. Poorer reflective functioning and increased symptoms of depression and anxiety will be related to reduced coparenting alliance for both parents.
2. Poorer reflective functioning will be related to increased symptoms of depression and anxiety for both parents.
3. There would be an indirect effect of maternal and paternal RF on coparenting relationships via parental symptoms of depression and anxiety.

### Design

**Figure 1:**
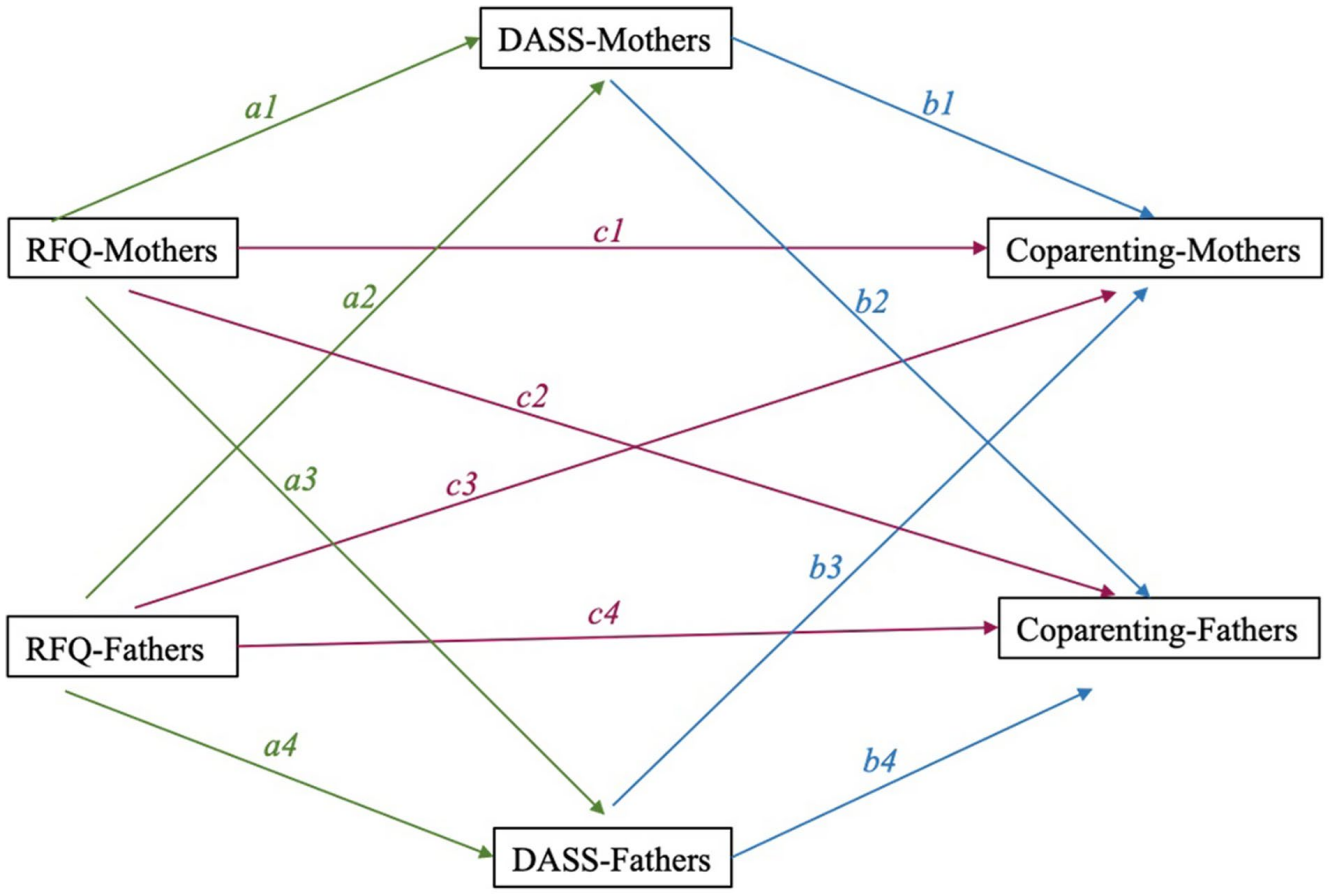
Relationship between reflective functioning, parental depression and anxiety and coparenting. RFQ = reflective functioning questionnaire, DASS = depression, anxiety and stress scale.

The present study implemented a cross-sectional, correlational research design to examine associations between parental mental health, parental reflective functioning (RF) and coparenting in couples. The researchers used this research design to test the following model (see Figure 1).

## Method

### Participants

Participants were 164 individuals representing 82 couples of a child aged 3 years to 17 years. Participants were recruited via Prolific, an online recruiting platform that renumerates participants for their time. Inclusion criteria were met if parents were in a mixed gender couple relationship and had a school attending child aged 3 to 17 years. To be eligible to participate both members of the couple were required to be Prolific users willing to participate in couple-based studies. Participants also needed to be proficient in the English language. While the data used in this study was part of a larger research project, participants were only included in the final analysis if data from both parents was available.

Participants’ ages ranged from 27 to 65 years (*M* = 41.24, *SD* = 5.37.24) and children were aged between 3 years 0 months and 17 years and 1 month (*M* = 109.81 months, *SD* = 44.68), with 50% identified to be female and 50% male. 80.5% of the sample were married, 3.7% were engaged, 15.9% were in a committed relationship. 73.2% of the sample were born in the United Kingdom, 11.6% were born in the United States of America, and the remaining participants were born in Bangladesh (1.2%), Canada (0.6%), China (1.8%), Germany (0.6%), Ireland (0.6%), Lithuania (0.6%), Nigeria (1.2%), Pakistan (1.2%), Poland (1.8%), Romania (1.2%), Saudi Arabia (1.2%), South Africa (1.8%) and Trinidad and Tobago (1.2%).

The sample was generally well-educated, with most participants (59.1%) having completed a tertiary degree (or higher). Most participants (91.5%) were employed, while the remaining 8.5% were either unemployed or unable to work for other reasons.

### Materials

The 21-item Depression Anxiety Stress Scale (DASS; Lovibond and Lovibond, 1995) asks participants to rate their symptoms of depression, anxiety and stress over the past 7 days, on a four-point Likert scale from 0 (‘Did not apply to me at all) to 3 (‘Applied to me very much, or most of the time’). Items include “I felt that I had nothing to look forward to” and “I felt scared without any good reason.” Each of the three 7-item subscales are then summed, with greater symptoms indicated by higher scores. This scale is widely used in clinical practice and research and has demonstrated excellent reliability and validity (Osman et al., 2012). In the present study the DASS-21 total score was used, and demonstrated excellent internal reliability (Cronbach’s *α* = 0.95). This scale has also shown good convergent validity, and is well correlated with other validated depression and anxiety measures (*r* = 0.5–0.8) (Osman et al., 2012).

The Brief Coparenting Relationship Scale (B-CRS; Lee et al., 2021) is a 12-item self-report questionnaire that measures the quality of coparenting interactions namely agreement, support, children’s exposure to parental conflict, undermining a parent’s role and overall coparenting quality. Items include “I believe my partner is a good parent” and “My partner undermines my parenting” and are rated on a 7-point scale from 0 (‘Not true of us’) to 6 (‘Very true of us’). To score, 5 items are reverse scored and then all items were summed with higher scores indicating a stronger coparenting quality. This is a reliable and valid tool (Lee et al., 2021), and was found to have good internal reliability in the present study (Cronbach’s *α* = 0.88). This scale was not found to be significantly different to the full scale CRS, with both demonstrating good construct validity (*r* = 0.60–0.74) as shown by correlations with related constructs including couple love and couple conflict (Feinberg et al., 2012; Lee et al., 2021). In the analysis, we used the total scale score rather than the subscales, in order to capture the information needed to test the model, i.e., a total coparenting relationship score for each parent.

The 8-item Reflective Functioning Questionnaire (RFQ; Fonagy et al., 2016) measures an individual’s RF on a 7-point Likert scale from 1 (‘do not agree at all’) to 7 (‘agree completely’). Items include “People’s thoughts are a mystery to me” and “I always know what I feel”. Originally this scale was divided into two subscales, certainty and uncertainty about mental states, however a recent validation study has argued that the scale is unidimensional, and has been found to reliably and validly measure uncertainty about mental states (Woźniak-Prus et al., 2022). Based on this study, the present study scored the RFQ-8 by taking an average of all items after reverse scoring item 7, with higher scores indicative of more uncertainty about mental states and poorer overall mentalizing capacity. In the present study internal reliability was found to be good (Cronbach’s α = 0.87) along with good construct validity (*r* = 0.39–0.66) as shown by correlations with related constructs including mindfulness and alexithymia (Cucchi et al., 2018).

### Procedure

Ethics approval was obtained from the University of Western Australia Human Research Ethics Committee (2023/ET000147). Following the Prolific recruitment process, participants accessed a Qualtrics survey via a link in the study advertisement. There participants read an explanatory statement, provided informed consent and completed all study measures which took on average 20 min to complete. Participants also provided their own and their partner’s unique Prolific ID so that responses could be matched.

Along with study measures, participants also answered several demographic questions (i.e., age, marital status and country of birth). To ensure items were responded to legitimately, an attention check question was included in the survey. Participants were credited £2.00 (approximately AU$3.80) for their time upon valid completion of the survey, and an additional £1.00 (approximately AU$1.90) bonus payment once their partner had also completed the survey.

### Data analysis plan

Analyses were run using SPSS (v.28). After evaluating correlations between study variables, the hypothesized indirect effects were tested using the Actor-Partner Interdependence Model extended to Mediation (APIMeM; see Figure 1). This analysis was done using the MEDYAD package in SPSS (Coutts et al., 2019), which takes into account the dependent nature of data from each member of a dyad by controlling for the other partner’s scores. This is done using an ordinary least squares (OLS) regression-based approach to mediation analysis. Bias-corrected bootstrapped confidence intervals (10,000 iterations; as recommended by Hayes, 2017) were used to test the indirect effect within the mother–father dyad of RF on coparenting via parental symptoms of depression and anxiety. This package does not test for model fit (which was also peripheral to the current study aims), but does allow us to identify whether there is a partial or full-indirect association between the hypothesised relationships between study variables.

## Results

The data in the results section is from the 82 couples described in the participants section.

### Assumption checks, bivariate correlations and descriptive statistics

The statistical assumptions underpinning regression-based methods (normality, linearity, homogeneity of variance, multicollinearity, and outliers) were assessed prior to the analysis. For both maternal and paternal cohorts, all but one variable was approximately normally distributed. The exception was fathers ratings on the DASS, which was slightly positively skewed, but still within acceptable limits. This was further supported by the normally-distributed, linear, and non-heterogeneity of variance in residual scores for both mother and father ratings. Data were also free from multicollinearity, evidenced by no exceedingly strong correlations between variables (see Table 1), and also tolerance values that fell above 0.2 and VIF scores that were less than 5.00. The data were also free from univariate outliers and multivariate outliers with high-leverage (e.g., Cook’s Distance exceeding 1.00). Accordingly, all relevant statistical assumptions were satisfied.

**Table 1:** Descriptive statistics and correlations between measurement variables (*N* = 164).

		Correlations		Descriptives		
	Variables	RFQ	DASS	*M*	*SD*	*α*
Combined	1. Reflective functioning	–	–	3.29	1.20	0.87
	2. DASS total	0.54^**^	–	22.31	21.55	0.95
	3. Coparenting total	−0.36^**^	−0.42^**^	59.60	10.50	0.88
Mothers	1. Reflective functioning	–	–	3.29	1.15	
	2. DASS total	0.53^**^	–	24.38	20.21	
	3. Coparenting total	−0.37**	−0.36^**^	59.39	10.52	
Fathers	1. Reflective functioning	-	–	3.29	1.25	
	2. DASS total	0.54^**^	–	20.24	22.75	
	3. Coparenting total	−0.35**	−0.46^**^	59.73	10.55	

Bivariate correlations are presented on the lower quadrant. **p < 0.001, *p < 0.05.

The bivariate correlations and descriptive statistics are provided in Table 1. All study variables were significantly correlated with each other (see Table 1). The correlation between each mother–father dyad’s scores on the DASS (*r* = 0.43, *p* < 0.001) and B-CRS (*r* = 0.65, *p* < 0.001) were strong, positive, and statistically significant. Scores on the RFQ for mother–father dyads were weak and not statistically significant (*r* = 0.13, *p* = 0.242). Potential covariates (total number of children, and age of youngest child) were only weakly and non-significantly correlated with maternal and paternal ratings across all study variables. Accordingly, they were not included in the final models.

The descriptive statistics suggest that the average DASS scores were below the clinically significant cutoffs for severe or extremely severe (Lovibond and Lovibond, 1995). However, mothers’ scores on the DASS were notably higher than fathers’ scores. In contrast to this, scores for reflective functioning and coparenting were extremely similar for mothers and fathers. The average of coparenting scores was moderately high, with the maximum score on this scale being 72, and the combined mean being 59.6 (Lee et al., 2021). The combined mean of reflective functioning scores was around average (3.29), with the scale having a range of 1 to 7 (Fonagy et al., 2016).

### Predicting mothers coparenting

The variables included in this APIMeM model accounted for a statistically significant 49% of the variance in mothers coparenting quality, equating to a large-sized effect. The total effect of mothers reflective functioning on mothers coparenting was statistically significant (*b* = −3.07, *p* = 0.001, 95% CI: −4.92, −1.22). The total effect of fathers reflective functioning on mothers coparenting was also statistically significant (*b* = −2.12, *p* = 0.015, 95% CI: −3.81, −0.42).

#### Predictors of mothers coparenting

In this APIMeM model, only mothers reflective functioning was found to significantly predict mothers coparenting quality (*b* = −2.49, *p* = 0.026, 95% CI: −4.66, −0.31). Fathers reflective functioning, and both mothers and fathers DASS scores were not found to be significant predictors of mothers coparenting quality. See Table 2.

**Table 2:** Predictors of coparenting in mothers, with 95% bias corrected confidence intervals reported in parenthesis.

Variables	*b*	*SE B*	*p*
C1 – Mothers Reflective Functioning	−2.49 (−4.66, −0.31)	1.09	**0.026**
C3 – Fathers Reflective Functioning	−1.09 (−0.09, 0.10)	0.99	0.277
B1 – Mothers DASS total	−0.05 (−0.19, 0.08)	0.07	0.449
B3 – Fathers DASS total	−0.09 (−0.21, 0.03)	0.06	0.136
Total effect of Mothers RFQ	−3.07 (−4.92, −1.22)	0.93	**0.001**
Total effect of Fathers RFQ	−2.115 (−3.81, −0.42)	0.85	**0.015**

Confidence intervals and standard errors based on 10,000 bootstrap samples (*N* = 82). *R*^2^ = 0.490. Bold values indicate statistically significant results.

### Predicting fathers coparenting

The variables included in this APIMeM model accounted for a statistically significant 51.6% of the variance in fathers coparenting quality, equating to a large-sized effect. The total effect of mothers reflective functioning on fathers coparenting was statistically significant (*b* = −1.94, *p* = 0.045, 95% CI: −3.83, −0.05). The total effect of fathers reflective functioning on fathers coparenting was also statistically significant (*b* = −2.68, *p* = 0.003, 95% CI: −4.42, −0.95).

#### Predictors of fathers coparenting

In this APIMeM model, only fathers DASS score was found to significantly predict fathers coparenting quality (*b* = −0.16, *p* = 0.009, 95% CI: −0.27, 0.04). Mothers and fathers reflective functioning, and mothers DASS scores were not found to be significant predictors of fathers coparenting quality. See Table 3.

**Table 3:** Predictors of coparenting in fathers, with 95% bias corrected confidence intervals reported in parenthesis.

Variables	*b*	*SE B*	*p*
C2 – Mothers Reflective Functioning	−1.29 (−3.44, 0.86)	1.08	0.235
C4 – Fathers Reflective Functioning	−1.01 (−2.96, 0.94)	0.98	0.306
B2 – Mothers DASS total	−0.051 (−0.19, 0.08)	0.07	0.452
B4 – Fathers DASS total	−0.16 (−0.27, 0.04)	0.06	**0.009**
Total effect of Mothers RFQ	−1.94 (−3.83, −0.05)	0.95	**0.045**
Total effect of Fathers RFQ	−2.68 (−4.42, −0.95)	0.87	**0.003**

Confidence intervals and standard errors based on 10,000 bootstrap samples (*N* = 82). *R*^2^ = 0.516. Bold values indicate statistically significant results.

### Predictors of mothers depression and anxiety scores

The variables included in this APIMeM model accounted for a statistically significant 57.7% of the variance in mothers’ depression, anxiety and stress symptoms, equating to a large-sized effect. Both mothers and fathers RF was found to significantly predict mothers DASS scores: Mothers RFQ (*b* = 9.08, *p* = 0.000, 95% CI: 5.84, 12.32), Fathers RFQ (*b* = 3.20, *p* = 0.035, 95% CI: 0.23, 6.17). See Table 4.

**Table 4:** Predictors of DASS in mothers, with 95% bias corrected confidence intervals reported in parenthesis.

Variables	*b*	*SE B*	*p*
A1 – Mothers Reflective Functioning	9.08 (5.84, 12.32)	1.63	0.000
A2 – Fathers Reflective Functioning	3.20 (0.23, 6.17)	1.49	0.035

Confidence intervals and standard errors based on 10,000 bootstrap samples (*N* = 82). *R*^2^ = 0.577.

### Predictors of fathers depression and anxiety scores

The variables included in this APIMeM model accounted for a statistically significant 54.6% of the variance in fathers’ depression, anxiety and stress symptoms, equating to a large-sized effect. Only fathers, but not mothers reflective functioning was found to significantly predict fathers DASS scores (*b* = 9.691, *p* = 0.000, 95% CI: 6.261, 13.122). See Table 5.

**Table 5:** Predictors of DASS in fathers, with 95% bias corrected confidence intervals reported in parenthesis.

Variables	*b*	*SE B*	*p*
A3 – Mothers reflective functioning	1.217 (−2.525, 4.960)	1.880	0.519
A4 – Fathers reflective functioning	9.691 (6.261, 13.122)	1.723	**0.000**

Confidence intervals and standard errors based on 10,000 bootstrap samples (*N* = 82). *R*^2^ = 0.546. Bold values indicate statistically significant results.

### Mediation analyses

Hypothesis 3. Tests of the indirect effect of RF on coparenting through symptoms of depression and anxiety are presented in Table 6. In the present study, there was an indirect effect of RF on coparenting through symptoms of depression and anxiety for fathers only (*b* = −1.509, 95% CI: −2.653, −0.513). Fathers’ ratings of their own RF (father actor effects) were significantly associated with their own symptoms of depression and anxiety (*b* = 9.691, *p* = 0.000, 95% CI: 6.261, 13.122) such that poorer RF was associated with more symptoms of depression and anxiety. Fathers’ self-reported symptoms of depression and anxiety were also significantly associated with fathers’ coparenting scores (*b* = −0.156, *p* = 0.009, 95% CI: −0.272, 0.040), while the direct association between fathers’ reflective functioning and fathers’ coparenting scores was not significant in the current model (*b* = −1.008, *p* = 0.979, 95% CI: −2.959, 0.941).

**Table 6:** Indirect effects of RF on coparenting via parental depression and anxiety, with 95% bias corrected confidence intervals reported in parenthesis.

Variables	*b*	*SE B*
Indirect pathway from RFQ to coparenting via DASS total for mothers	−0.471 (−1.828, 0.924)	0.679
Indirect pathway from RFQ to coparenting via DASS total for fathers	−1.509 (−2.653, −0.513)	0.548

Confidence intervals and standard errors based on 10,000 bootstrap samples (*N* = 82).

**Figure 2:**
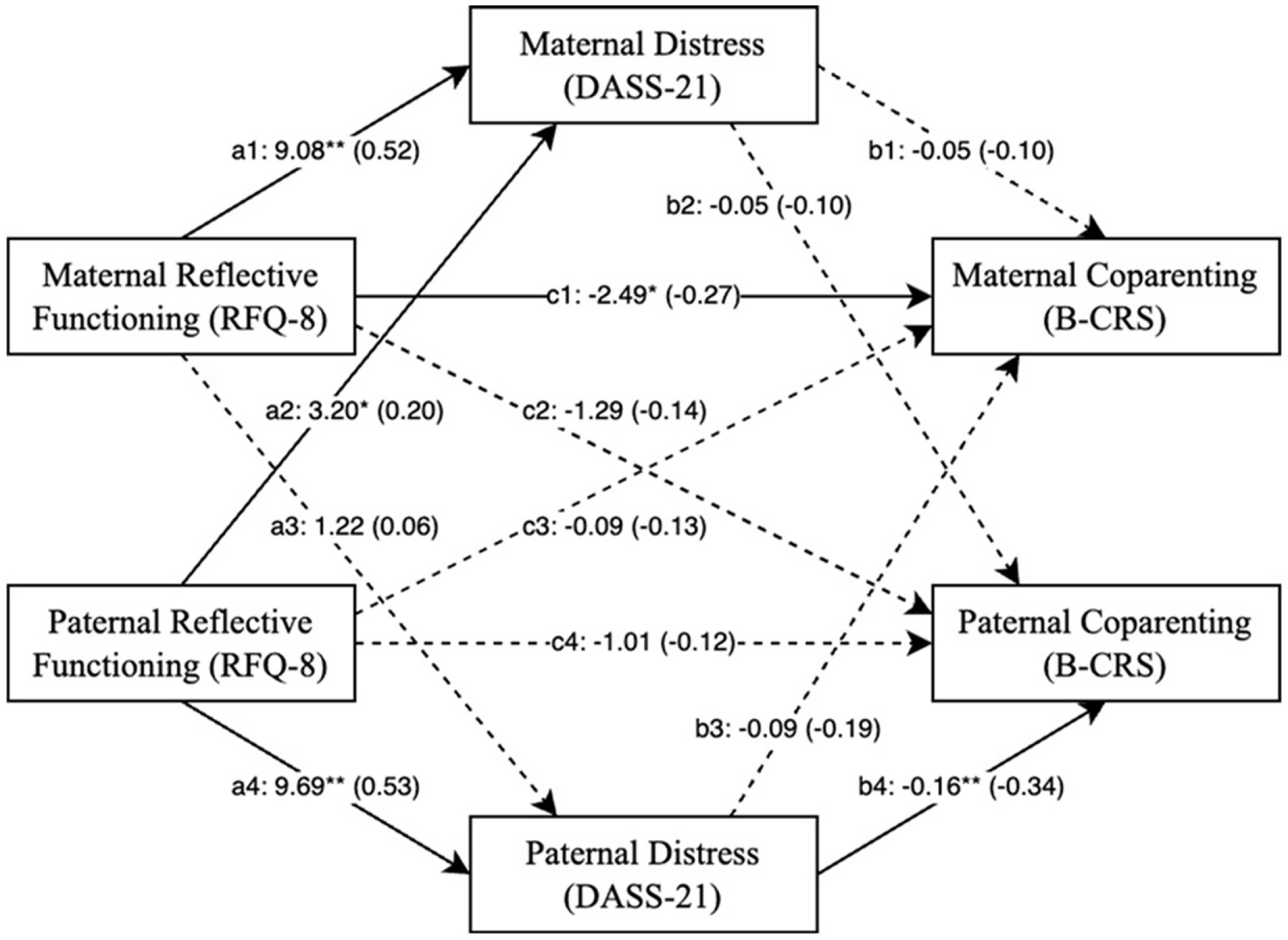
Relationship between reflective functioning, parental depression and anxiety and coparenting and child social emotional symptoms with standardised coefficients. RFQ = reflective functioning questionnaire, DASS = depression, anxiety and stress scale, * = *p* < 0.05, ** = *p* < 0.01.

As with fathers, mothers’ RF (mother actor effects) was significantly associated with symptoms of depression and anxiety (*b* = 9.078, *p* = 0.000, 95% CI: 5.838, 12.319) such that poorer RF was associated with more symptoms of depression and anxiety. However, for mothers, symptoms of depression and anxiety did not directly influence their coparenting scores (*b* = −0.052, *p* = 0.068, 95% CI: −0.188, 0.084), and a direct association between mothers RF and their coparenting quality was found (*b* = −2.487, *p* = 0.026, 95% CI: −4.662, −0.312), with no indirect effect present (*b* = −0.471, 95% CI: −1.828, 0.924). See Table 6.

## Discussion

The overall aim of the present study was to cross-sectionally investigate relationships between general RF, parental depression and anxiety, and coparenting among couples.

As per hypothesis one, we found that poorer RF in mothers was related to reduced coparenting quality. This relationship aligns with previous research (De Palma et al., 2023; Holtzinger, 2021; Jessee, 2012; Marcu et al., 2016; Shai et al., 2017) and theoretical frameworks, suggesting that when parents can understand both their own and their partner’s internal worlds, communication improves, conflict may decrease, and parents are better able to collaborate effectively as a team (Borelli et al., 2020). However, we did not find this same link between RF and coparenting for fathers. Prior research examining gender differences within this relationship has been mixed, with one study finding a stronger relationship between RF and coparenting quality among fathers compared with mothers (Holtzinger, 2021), while another study found an association between improved coparenting quality and greater RF only for fathers with daughters and mothers with sons (Jessee, 2012). One possible reason for the gender difference found within the current research is the presence of an indirect effect for fathers only, whereby RF indirectly predicted coparenting via symptoms of depression and anxiety. Thus, the direct relationship between RF and coparenting was no longer significant when symptoms of depression and anxiety were considered in the model, whereas for mothers no indirect effect was found, and the direct effect remained significant.

As per the second hypothesis, we found a relationship between increased symptoms of depression and anxiety and reduced coparenting quality. However, this finding was only significant for fathers. This relationship was expected given prior research indicating that parental depression and anxiety may predict poor coparenting relationships (Turney and Hardie, 2021; Williams, 2018). This can be understood by considering the significant impact mental health has on general functioning. In the present study it is not clear why this relationship is present only for fathers. It may be that with all variables entered into the model this relationship was no longer significant for mothers, whereas other studies examined only coparenting and mental health and did not consider the role of RF. Prior literature that has examined gender differences in relation to these variables is mixed, with some studies finding no gender differences (Metz et al., 2018; Turney and Hardie, 2021), while other studies did for either mothers or fathers only (Tissot et al., 2017; Williams, 2018). It is also interesting to note that in the present study, no significant relationships were found between fathers’ mental health and mothers’ coparenting, or vice versa. Prior research has found a relationship between fathers’ depression and mothers coparenting alliance (Williams, 2018), and this relationship would make sense given conceptual links between these constructs. For example, you may expect that if one parent is struggling with their mental health it may make it difficult for both members of a couple to work well as a parenting team. It is unclear why this was not found to be the case in the present research. Once again this may be because other variables in the model were more strongly linked, thus when considering the model as a whole these relationships became less important. It is also likely that these relationships are bidirectional, whereby struggling to work together in a coparenting team may also worsen mental health for fathers (Williams, 2018).

Hypothesis three considered the relationship between RF and parental mental health. In line with prior research (Belvederi Murri et al., 2017; Bigelow et al., 2018; De Palma et al., 2023; Wendelboe et al., 2021), we anticipated that there would be a significant relationship between RF and parental depression and anxiety, and this was found to be the case. In both mothers and fathers, increased symptoms of depression and anxiety were found when parents displayed poorer RF. This relationship makes sense conceptually given the role RF plays in regulating emotions and forming stable relationships, both factors that have been consistently linked with mental health (Luyten et al., 2013; Luyten and Fonagy, 2018). In the present study there was a significant relationship between fathers’ RF and mothers’ mental health, while mothers’ RF was not significantly related to fathers’ mental health.

This is possibly due to the gender roles of mothers and fathers within the parenting relationship. A study found that mothers tend to place increased value on their parenting role, with their identity more closely linked to their performance as a mother, while fathers’ identities are more linked to their role as a provider or the way that they contribute to society (Bonhag and Froese, 2022; Kaźmierczak and Karasiewicz, 2019). The literature also indicates that mothers undergo a complex identity shift when they have children, which is lifelong and may involve having a significantly different role in society, as well as relationships and home life (Kaźmierczak and Karasiewicz, 2019; Ruppanner et al., 2019; Umberson et al., 2010). Thus, it is likely that women feeling understood by their partners in their role as a mother is a significant influencing factor for mental health, whereas different factors may play a part for paternal mental health, such as their role in the more external community (Bonhag and Froese, 2022). With this in mind, fathers’ capacity to hold the mother’s mental state in mind may be particularly significant for maternal mental health, over and above the role that mothers’ RF may play in fathers’ mental health.

In the present study we also found an indirect effect of reflective functioning on the coparenting relationship via symptoms of depression and anxiety. Similar to the second hypothesis, this association was only found for fathers. We tested for this relationship based on consistent links in the literature between reflective functioning, parental mental health, and coparenting, and emerging research indicating that reflective functioning and coparenting may also be related (De Palma et al., 2023). In mothers the direct relationship between reflective functioning and coparenting remained significant, while the relationship between mental health symptoms and coparenting was not significant. For fathers the inverse was true. This suggests that for mothers the direct relationship between reflective functioning and coparenting is more important, and the role of depression and anxiety is secondary. For fathers the opposite appears true, with paternal mental health playing a significant role in the coparenting relationship.

For mothers it appears that capacity to hold their partner’s mind in mind is significantly and directly related to their ability to work together as a coparenting team regardless of maternal mental health. Again, this difference may be due to gender roles of mothers and fathers (Henderson et al., 2016). Research suggests that women perceive their significance in the world based largely on interpersonal relationships, such as parenthood and romantic relationships (Bonhag and Froese, 2022). This could mean that, due to the importance of close family relationships to a mother’s identity, she is more likely to either bypass or effectively manage her own mental health in the interest of the coparenting relationship and family wellbeing. On the other hand, in fathers the relationship between reflective functioning and coparenting was more significantly impacted by fathers’ mental health. While depression and anxiety in mothers tends to be more widely acknowledged and supported, there is much less awareness of poor mental health in fathers (Edward et al., 2019). This may lead to less access to helpful coping skills. Fathers may also be less willing to engage in help-seeking behaviour, as reflected in the literature (Sharp et al., 2023). Research also suggests that men are more likely to base their significance in the world on external factors, such as their roles in the wider community (Bonhag and Froese, 2022) and thus may not have the same motivations as mothers for managing their mental health. Despite these gendered differences, it is clear that for both mothers and fathers reflective functioning plays a significant role in both parental mental health and the coparenting relationship.

### Strengths, limitations and future directions

Using a sample of 82 mother–father dyads (164 individuals) allowed our study to effectively test the associations between variables. However, to accomplish this, we had to impose a wider age range (i.e., parents of children aged 3 to 17 years). This may influence the overall interpretability of our findings, as well as implications for tailored and targeted intervention. However, highlighting these relationships across stages of child development might also identify the importance of parental reflective functioning, parent mental health, and the coparenting relationship throughout developmental stages. It is possible that other factors (e.g., managing children with challenging behaviours) could also be relevant in better understanding what shapes the coparenting relationship. Future studies could include measures related to child functioning to better contextualise the association between individual parents, the parental alliance, and their children’s functioning.

Our study is further strengthened by our use of an Actor-Partner Interdependence Model, which allows us to consider the dependent nature of data from each parent by controlling for the other partner’s scores. Despite these strengths, several limitations should be considered. Given the cross-sectional nature of the current data, causal inferences cannot be drawn. While our model was informed by theory and prior research, each variable included is likely to have a bidirectional relationship with other study variables. While both literature and theory suggest that low mentalizing capacity may predict poor mental health outcomes, evidence also suggests that when mental health is poor, mentalizing capacity will worsen (Luyten et al., 2013; Luyten and Fonagy, 2018). The relationship between coparenting and parental mental health is similarly reciprocal. Research suggests that parental depression and anxiety may cause problems in the coparenting relationship, however the inverse may also be true, with research finding that poor coparenting may lead to increased parental depression and anxiety (Cabrera et al., 2012). This is important to consider when interpreting the present findings, however we feel that the current study remains relevant given the dearth of research examining these variables together. This field of research would benefit from additional longitudinal studies to assist in clarifying the direction of relationship between reflective functioning, parental mental health and coparenting.

Future studies could aim to recruit not only a larger number of participants, but a range of parents with different demographic backgrounds. For example, the majority of the current sample were educated, employed, and born in a Western country, so it would be beneficial to recruit more couples from non-Western cultures. There are also different gender roles and parenting practices across cultures which could influence results.

Our study is also limited by the self-report nature of study variables. Both RF and coparenting quality may be measured more accurately through observation or interview tasks which may provide a more rich and complete picture. Short self-report measures may miss nuance, and are open for reporter bias. Future research may benefit from including data from direct observation and parent interviews. We also did not screen for participant fraud, which presents a limitation for the validity of the data (Nur et al., 2024).

Floor and ceiling effects may also be important to consider within the current study (Hessling et al., 2004), with generally low average scores on the DASS (which measure depression, anxiety and stress), and high average scores on the BCRS, which suggests that a large proportion of the sample had few mental health concerns and a generally strong coparenting quality. The majority of study participants were born in Western countries, and therefore these findings may be less applicable to other cultural contexts. The relationship between RF and coparenting would benefit from further investigation in other cultural contexts.

Our use of the prolific platform for participant recruitment could be considered a limitation, as participants complete surveys online and are reimbursed for their time. This could mean that the participants in the study tended to be couples who were needing money and had access to the internet. Couples who were struggling with their mental health or with parenting would also be less likely to complete a survey of this nature, which may also relate to the floor and ceiling effects just discussed.

The cross-sectional nature of the study should also be noted as a limitation. A cross-sectional study design limits the ability to report and establish causality, therefore, only associations can be identified. In addition, the study lacks temporal sequencing, where it is not made clear the direction of the relationships. Furthermore, confounding variables are not taken into account and therefore may influence the variables.

### Implications

This study contributes to a small body of research examining the relationship between RF and coparenting among parents. The present study also considered the role of parental mental health and how these variables relate among mother–father dyads. As with prior research, the present study once again indicates that these variables are strongly linked. This is some of the first research to consider how gender impacts these variables among couples. Because parental mental health, coparenting and RF have each been consistently linked with child development and outcomes later in life, it is important to consider how these variables interact with one another. Understanding these interactions may inform interventions that target parent and child wellbeing. It is clear from these findings that interventions targeting reflective functioning will benefit both mothers and fathers, potentially improving mental health for both while also enhancing the coparenting relationship. We suggest incorporating content on reflective functioning into couples therapy, and also parenting classes for new couples. Previous research has investigated the results of programs that target reflective functioning in the parent–child dyad; however there is scope for the further development of programs that target reflective functioning in the couple relationships (Barlow et al., 2020).

In line with prior research, our findings show that RF is strongly linked to both parental mental health and the coparenting relationship. We hope these findings will inform future research in this important field, and encourage more interventions for parents to consider mentalizing capacity as important targets for change. Our research suggests that targeting RF may improve parental mental health, specifically symptoms of anxiety and depression, and enhance the quality of coparenting of parents. When the family unit is functioning well, infants and children have the greatest chance to develop optimally and long-term child outcomes may be improved.

## Data Availability

The raw data supporting the conclusions of this article will be made available by the authors, without undue reservation.
